# The impact of intensive trauma-focused treatment on sexual functioning in individuals with PTSD

**DOI:** 10.3389/fpsyg.2023.1191916

**Published:** 2023-08-08

**Authors:** Clair van Woudenberg, Eline M. Voorendonk, Bo Tunissen, Vince H. F. van Beek, Linda Rozendael, Agnes Van Minnen, Ad De Jongh

**Affiliations:** ^1^Research Department, PSYTREC, Bilthoven, Netherlands; ^2^Behavioural Science Institute (BSI), Radboud University Nijmegen, Nijmegen, Netherlands; ^3^Research Department, PSYTREC, Weert, Netherlands; ^4^Academic Centre for Dentistry Amsterdam (ACTA), University of Amsterdam and VU University Amsterdam, Amsterdam, Netherlands; ^5^School of Psychology, Queen’s University, Belfast, United Kingdom; ^6^Institute of Health and Society, University of Worcester, Worcester, United Kingdom

**Keywords:** post-traumatic stress, PTSD, sexual functioning, sexual satisfaction, sexual desire, intensive trauma-focused treatment, EMDR therapy, prolonged exposure therapy

## Abstract

**Background:**

Individuals with posttraumatic stress disorder (PTSD) often experience sexual disturbances.

**Objective:**

To determine whether intensive trauma-focused treatment is associated with an improvement in sexual functioning (i.e., sexual satisfaction and sexual desire) in individuals with PTSD.

**Method:**

In total, 227 patients with PTSD (68.7% women, mean age = 40.97) participated in an intensive eight-day trauma-focused treatment program consisting of prolonged exposure, eye movement and desensitization and reprocessing (EMDR) therapy, physical activity, and psychoeducation. Patients were assessed (i.e., Clinician Administered PTSD Scale and Sexual Functioning Questionnaire) pre- and post-treatment and at 6-months follow-up.

**Results:**

Sexual satisfaction and sexual desire increased significantly associated with trauma-focused treatment from pre-treatment to 6-months follow-up, albeit the effect sizes were small (Cohen’s *d =* 0.39 and 0.17, respectively). Although men reported greater overall sexual desire than women, sexual functioning improved after treatment in both men and women. Furthermore, those with remission of PTSD reported greater sexual functioning post-treatment and at 6-months follow-up, than those without remission. However, changes in PTSD symptoms associated with treatment were not predictive of the level of sexual satisfaction or sexual desire 6 months after treatment.

**Conclusion:**

The results of this uncontrolled study suggest that intensive treatment for PTSD can have beneficial effects on sexual satisfaction and desire in both men and women; however, this may not necessarily be due to a decrease in PTSD symptoms.

## Introduction

Post-traumatic stress disorder (PTSD) is a common and often disabling mental disorder with a high comorbidity rate (Brady et al., 2000). One category of comorbid conditions frequently reported by those with PTSD is sexual dysfunction (Yehuda et al., 2015). For example, in male veterans with PTSD, sexual dysfunction is observed in 60–85% of cases (Badour et al., 2015; Bentsen et al., 2015). In a sample of female PTSD patients with childhood sexual abuse, 81% reported difficulties in engaging in sexual activities (Kratzer et al., 2022). PTSD has been found to have a negative association with overall sexual functioning, sexual satisfaction, sexual desire, and sexual distress. Conversely, the results were mixed for sexual arousal, orgasm function, erectile dysfunction, premature ejaculation, sexual pain, and the frequency of sexual activity (Bird et al., 2021).

Several factors have been suggested as potential explanations for the high prevalence of sexual dysfunction in individuals with PTSD, including comorbid mood disorders (Clayton et al., 2014; Yehuda et al., 2015), the use of psychopharmaceuticals (Chiesa et al., 2013), and exposure to sexual assault (Najman et al., 2005). However, it has been suggested that, regardless of the type of trauma history, dysfunctions are primarily associated with the mere presence of PTSD symptoms (Yehuda et al., 2015; Bornefeld-Ettmann et al., 2018). This notion is supported by the frequent presence of sexual problems and decreased sexual satisfaction in combat veterans and ex-prisoners of war who generally did not experience sexual violence (Zerach et al., 2010; Hosain et al., 2013; Lehrner et al., 2016). Regarding female veterans, a study showed that the type of trauma experienced was not associated with differences in sexual functioning. However, sexual trauma was found to be related to more frequent PTSD symptoms, which were subsequently associated with reduced sexual functioning (DiMauro et al., 2018). This suggests that the relationship between sexual trauma and sexual problems might be moderated by the severity of the PTSD symptoms associated with sexual trauma. Similarly, Schnurr et al. (2009) found that the severity of PTSD symptoms was correlated with dysfunctional sexual behavior and sexual concerns in female veterans.

Despite its high prevalence in individuals with PTSD, the presence of sexual dysfunction is not included as a criterion in the DSM-5 classification of PTSD and appears to be rarely targeted within trauma-focused treatment protocols (O'Driscoll and Flanagan, 2016). This might be due to the fact that sexual dysfunction is often not presented as a primary complaint by those suffering from PTSD (Tran et al., 2015; Yehuda et al., 2015). Nevertheless, sexuality is considered an essential and integral part of most interpersonal relationships (Dewitte, 2012; Yehuda et al., 2015). Most adults report the importance of healthy sexual functioning and pleasurable sex to their quality of life (Flynn et al., 2016), which is also the case for those suffering from PTSD (Nunnink et al., 2012). Given that greater adaptive sexual functioning has been associated with better physical and psychological health (Scott et al., 2012), and reduced suicide risk in male veterans with PTSD (Khalifian et al., 2020), adequate treatment of sexual dysfunction is important.

Assuming that sexual dysfunction largely depends on the presence of (symptoms of) PTSD, one might question whether these dysfunctions should be treated separately or whether trauma-focused treatment would also alleviate sexual dysfunction by its effect on PTSD symptom severity. The results of studies investigating this relationship remain somewhat ambiguous (Schnurr et al., 2009; Tarquinio et al., 2012; Yehuda et al., 2015), with studies showing improvement in symptoms of sexual dysfunction (Resick et al., 2003; Wells et al., 2019; Badour et al., 2020), and studies showing no improvement at all (e.g., O'Driscoll and Flanagan, 2016). A possible explanation for the lack of improvement in sexual functioning in individuals with PTSD (O'Driscoll and Flanagan, 2016) is that evidence-based trauma-focused treatments are still rarely applied (Van Minnen et al., 2010; Smith et al., 2020). To date, the effects of PTSD treatment on sexual functioning have been studied almost exclusively in women with PTSD. Therefore, further studies of the effects of evidence-based trauma-focused therapy on sexual dysfunction in both women and men are warranted.

The purpose of the present study was to determine the effects of brief intensive trauma-focused treatment on sexual functioning for individuals suffering from PTSD. As sexual desire and pleasure (i.e., satisfaction) correspond to the two main factors of the human sexual response cycle (Masters and Johnson, 1966) and have been found to be negatively associated with the presence of PTSD (Bird et al., 2021), we were particularly interested in these two outcome variables. The sample consisted of men and women, mostly civilians, who had been exposed to a wide range of traumatic events. Given the established effectiveness of intensive treatment programs on symptoms of PTSD (Van Woudenberg et al., 2018; Sciarrino et al., 2020; Burback et al., 2023), and findings suggesting that sexual dysfunction is related to the presence of PTSD symptoms, we hypothesized that sexual satisfaction and sexual desire would significantly improve with the application of a brief intensive trauma-focused treatment program lasting 8 days. In addition, we examined potential differences in treatment effects regarding sexual functioning between men and women, those with remission and no remission of PTSD following treatment, and the relationship between the effect of the treatment program on PTSD symptoms on the one hand, and sexual functioning at follow-up on the other.

## Materials and methods

### Participants

A total of 428 participants were recruited from two treatment locations in the Netherlands (PSYTREC) and included in this study. At this facility, patients undergo an inpatient trauma-focused treatment program, which consists of 4 days of treatment, a weekend at home and another 4 days of treatment. Patients were referred by their general practitioners, psychologists, or psychiatrists. The inclusion criteria for the treatment program were as follows: (1) being at least 18 years old, (2) having sufficient knowledge of the Dutch language, and (3) having been diagnosed with PTSD according to the DSM-5. Patients were excluded from treatment in case of (1) a recent suicide attempt (within 3 months prior to intake) and (2) being convicted for sexual violence in their past. Measurements were provided as part of routine treatment outcome measurements; no (financial) compensation was offered. This study was conducted in accordance with the principles of the Declaration of Helsinki. According to the Medical Ethics Review Committee of VU University Medical Centre (registered with the US Office for Human Research Protections (OHRP) as IRB00002991, FWA number FWA00017598), the Medical Research Involving Human Subjects Act (WMO) does not apply to this study. For this reason, the committee did not require the official approval of this study. The patient did not receive any financial compensation.

**Figure 1:**
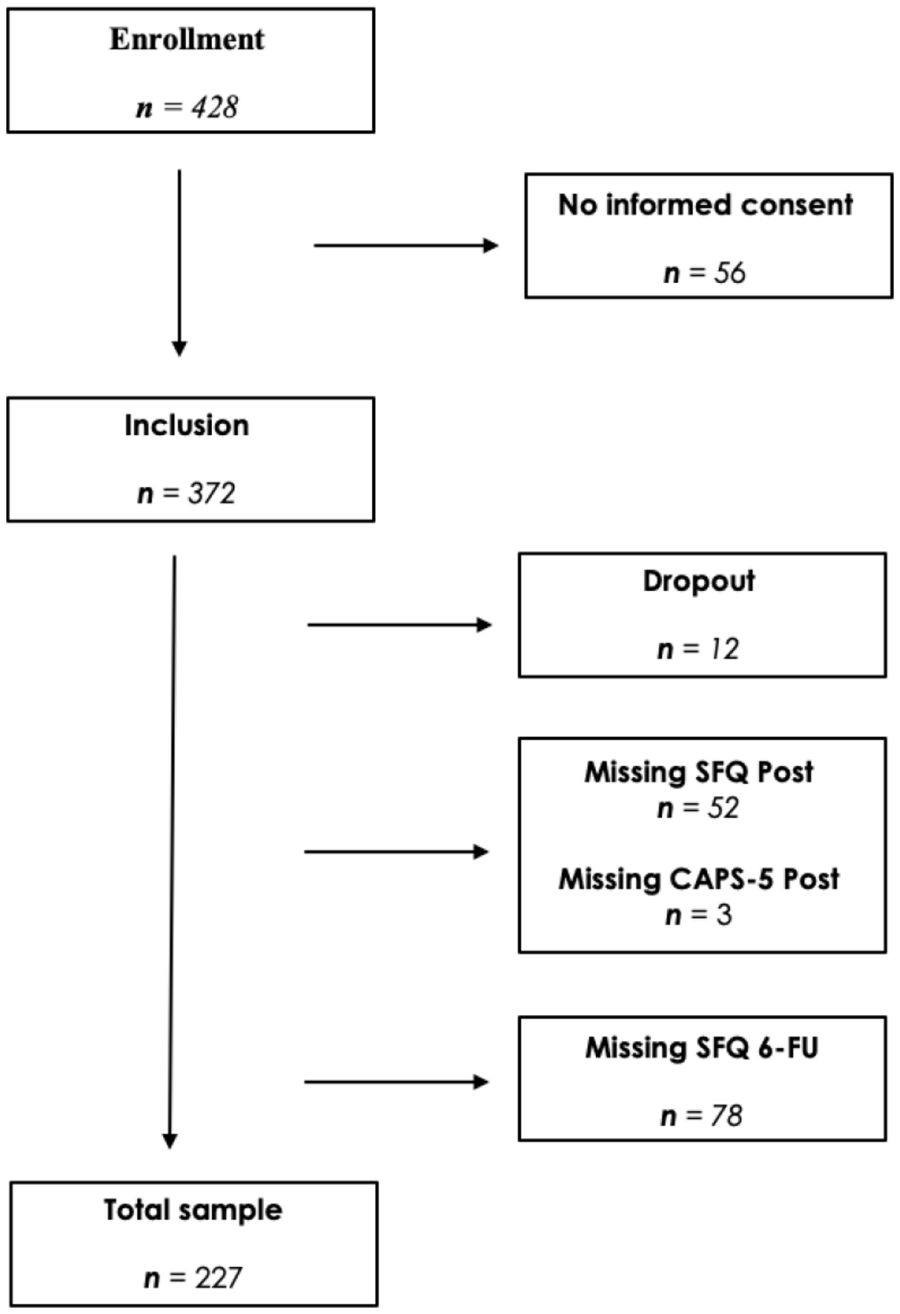
Flowchart of participants. Clinician Administered PTSD Scale for DSM-5 (CAPS-5); Sexual Functioning Questionnaire (SFQ).

Informed consent was obtained from each patient before inclusion in the study. A total of 372 (59.4% female) participants with a mean age of 40.11, gave informed consent and were included. Twelve participants dropped out of the treatment. Furthermore, owing to missing data on post-treatment measurements (*n* = 55) or follow-up measurements (*n* = 78), 133 participants were excluded prior to the analyzes. The final sample consisted of 227 patients. The flow of participants is shown in Figure 1.

#### Measures

The demographic characteristics of the treatment sample were gathered prior to the start of treatment using self-report demographic questionnaires at the treatment centre.

*The Sexual Functioning Questionnaire* (SFQ) is a self-report measure used to assess sexual functioning (see Appendices for the Dutch version and the English translation) constructed for the present study. The questions were extracted from the Female Sexual Functioning Index (FSFI; Rosen et al., 2000) and the International Index of Erectile Function (IIEF; Rosen et al., 1997). As no validated Dutch versions of these questionnaires were available, the items were translated from English to Dutch and back-translated to identify any discrepancies, differences in meaning, or other potential issues. The SFQ included four items assessing relationship status (item 1; having a partner, yes or no), the frequency of sexual intercourse or masturbation in the previous month/week [item 2; rated at a 6-point ordinal scale; scored as none (1), 1–2 times (2), 3–4 times (3), 5–6 times (4), 7–10 times (5), and 11 times or more (6)], the amount of satisfaction that was associated with sexual intercourse or masturbation in the past month/week [item 3; rated at a 5-point scale; no satisfaction (1), little satisfaction (2), moderate satisfaction (3), great satisfaction (4), and really great satisfaction (5)], and the frequency of subjective sexual desire [item 4; rated at a 5-point ordinal scale; never or almost never (1), sometimes (2), half of the time (3), often (4), and always or most of the time (5)]. Patients were instructed to only fill out the third item (sexual satisfaction) in case of sexual intercourse or masturbation in the past month, meaning a score of 2 or higher on item 2. At pre-treatment and at the 6-months follow-up, a past-month version of the SFQ was used, whereas at post-treatment, a past-week version was used. Cronbach’s alpha of the SFQ at pre-treatment in the current sample was 0.73.

*The Clinician-Administered PTSD Symptom Scale for DSM-5* (CAPS-5: Weathers et al., 2018; Dutch version Boeschoten et al., 2018) was used to assess PTSD diagnosis and symptom severity. The CAPS-5 is a widely used clinician-administered structured interview to assess PTSD symptoms according to the classification system of the DSM-5. It is considered the gold standard for PTSD assessment (Weathers et al., 2018). The CAPS-5 symptom items have a 5-point severity scale (0 = absent, 1 = mild/subthreshold, 2 = moderate/threshold, 3 = severe, 4 = extreme), with a total score ranging from 0 to 80. The SEV2 rule, in combination with the DSM-5 algorithm, was used to diagnose PTSD (Weathers et al., 2013; Boeschoten et al., 2018). Patients are regarded as ‘in remission’ if there is no longer a PTSD diagnosis and if they have a total CAPS-5 score of 12 or lower post-treatment. The Dutch version of the CAPS-5 has been found to be a valid and reliable measure with a Cronbach’s alfa of 0.90 and a high inter-rater reliability of 0.98 (Boeschoten et al., 2018).

Traumatic exposure was assessed using the *Life Event Checklist for DSM-5* (LEC-5: Weathers et al., 2013). This 17-item self-report measure assesses the number and type of traumatic events experienced by a person during their lifetime. These items encompass several possible traumatic events (e.g., natural disasters, physical violence, sexual assault, exposure to toxic substances, and combat). In the present study, the Dutch translation of the LEC-5 was used (Boeschoten et al., 2014). The original LEC has been found to be a reliable measure of traumatic events (Gray et al., 2004).

The Dutch version of the *Mini International Neuropsychiatric Interview* (MINI-Plus; Sheehan et al., 1998; Overbeek et al., 1999) was used to assess comorbid psychiatric disorders and suicide risks. The MINI-plus is a widely used, structured diagnostic psychiatric interview with good psychometric characteristics that has been found to be useful in diagnosing psychiatric patients (Van Vliet and De Beurs, 2007). For the purpose of this study, only sections assessing anxiety disorders, mood disorders, and suicide risk were used.

#### Procedure

The inclusion and exclusion criteria were checked during the diagnostic phase, which consisted of two intake sessions. Subsequently, traumatic experiences, PTSD severity, and psychiatric comorbidity were assessed by a clinician using the LEC-5, CAPS-5, and MINI-plus. If the inclusion criteria were met, patients were invited to undergo treatment. Informed consent was obtained for inclusion in this study. The SFQ was administered by computer-assisted web interviewing (CAWI; https://www.idsurvey.com/en/cawi-methodology) between the two intake sessions and completed by the patients on their personal computer. Eight days after the last treatment day, the patients were invited to undergo a post-treatment assessment of PTSD symptoms at one of the facilities using the past-week version of the CAPS-5. The SFQ was administered again using CAWI and was completed using a personal computer. Six months after treatment, the SFQ was administered again using this mode.

#### Treatment

The intensive trauma-focused treatment program (for a description see Van Woudenberg et al., 2018) consisted of 16 (90-min) sessions with two different protocolled evidence-based therapies, carried out twice for four consecutive days of treatment, during which patients stayed at one of the locations. The patients were not taught stabilization (i.e., emotion regulation) techniques prior to treatment (for rationale, see De Jongh et al., 2016). Patients received two individual therapy sessions per day, provided by a certified and trained psychologist, lasting one and a half hours: prolonged exposure (PE) and eye movement desensitization and reprocessing (EMDR) therapy. The protocol used for PE (Foa et al., 2007) consisted of a structured combination of imaginal exposure and *in vivo* exposure. Patients were instructed to describe their traumatic memories as vividly and as detailed as possible out loud in the present tense. To test specific harm expectancies or to maximize fear activation, *in vivo* materials, such as sound fragments, pictures, or video clips were added during imaginal exposure. The standard EMDR protocol was used (Shapiro, 2018; De Jongh and Ten Broeke, 2019). In line with Baddeley’s (2012) working memory theory, working memory taxation during EMDR therapy was achieved using several tasks, specifically eye movements (following fingers or a light bar), which were combined with auditory tasks (listening to beeps), spelling tasks, vibrating handpieces, or other typical tasks that tax working memory (see Houben et al., 2020), all parts of the 2.0 version of EMDR therapy (Matthijssen et al., 2021). Treatment-interfering anticipatory fear was addressed using the flashforward protocol (Logie and De Jongh, 2014).

A therapist rotation system was applied (Van Minnen et al., 2018), and the patients received individual therapy from a different psychologist in each session. In addition to the individual therapy sessions, patients participated in six group psychoeducation sessions each day with the aim of gaining insight into their own PTSD symptomatology and treatment plan (Pratt et al., 2005). Another component of treatment is physical activity. Patients engaged in 6 hours of physical activity each day, both indoors and outdoors, which consisted of both low- and high-intensity activities (e.g., mountain biking, obstacle course, walking, and trust-focused activities). The rationale underlying the physical activity component of the treatment program has been well-described by Voorendonk et al. (2022).

#### Data analyzes

Statistical analyzes were performed using IBM SPSS 27 (IBM SPSS version 27 for Macintosh. IBM Corporation, Armonk, NY, United States). Descriptive analyzes (frequencies and means) were performed to assess pre-treatment patient characteristics. The data were screened for outliers and violations of assumptions. First, independent sample *t-*tests were performed to compare the baseline scores of participants with missing post-treatment and/or follow-up data with those in the final sample. No significant differences were found in the baseline CAPS or SFQ scores. A paired sample *t*-test was performed to establish the effect of treatment on PTSD symptoms. Since participants were instructed to only fill out Item 3 of the SFQ (sexual satisfaction) if they reported sexual intercourse or masturbation (Item 2) in the past month or week, only those who filled out this item were included in the analyzes concerning sexual satisfaction. One-way repeated-measures ANOVAs were performed to assess the effects of the treatment on sexual functioning (i.e., sexual satisfaction and sexual desire) from pre-treatment to post-treatment to the 6-months follow-up. Mixed model repeated measures ANOVAs were used to test whether there was an interaction between changes over time (pre- to post-treatment to 6-months follow-up) in both sexual satisfaction and sexual desire for men and women, as well as for those with post-treatment remission of PTSD and those without. Moreover, multiple linear regression analyzes were conducted to determine whether changes in CAPS-5 scores from pre- to post-treatment predicted sexual functioning scores at the follow-up. Effect sizes from pre- to post-treatment and from pre-treatment to the 6-months follow-up were calculated using Cohen’s *d* to facilitate comparability. For all analyzes, a two-tailed statistical threshold of *α* < 0.05 (two-tailed) was adopted.

## Results

### Baseline characteristics

In total, 227 participants were included in the analysis. The baseline characteristics of the study population are shown in Table 1. The mean age of the study sample was 40.97 (*SD* = 12.85) and ranged from 18.73 to 72.28, with 68.7% of the participants being female. On average, the study sample had a mean CAPS-5 score of 42.68 (*SD* = 7.52) at baseline. Most participants reported having been exposed to multiple types of traumatic events, such as sexual and/or physical abuse, motor accidents, and medical trauma. At baseline, 58.1% of the participants were diagnosed with at least one comorbid mood disorder, and suicide risk was moderate or high in up to 32.2% of the participants. Baseline sexual functioning scores are presented in Table 2.

**Table 1:** Baseline sample characteristics (*N* = 227).

	Frequency (%)
Gender (% female)	159 (68.5)
Trauma exposure	
Sexual abuse	201 (88.5)
Physical abuse	213 (91.8)
Major (motor) accident	125 (55.1)
War trauma	17 (7.5)
Life threatening illness or injury	70 (30.8)
Other trauma*	140 (61.7)
Comorbidity**	
Mood disorder	132 (58.1)
Anxiety disorder	99 (43.6)
Psychotic disorder	20 (8.8)
Suicidal risk	
None-Low	149 (65.6)
Moderate-High	73 (32.2)
Current relationship	137 (60.4)

*Other traumas include natural disasters and medical trauma; **MINI-Plus data were missing for 5 participants.

**Table 2:** Descriptives of sexual functioning for the total sample.

	Pre-treatment	Post-treatment	Follow-up
	Frequency (%)	Frequency (%)	Frequency (%)
**Current relationship**	137 (60.4)	141 (62.1)	139 (61.2)
Sexual intercourse and/or masturbation in the past month*	132 (58.1)	119 (52.4)	146 (64.3)
**Sexual satisfaction**			
No	19 (14.4)	9 (7.6)	10 (6.8)
Little	31 (23.5)	16 (13.4)	28 (19.2)
Moderate	45 (34.1)	45 (37.8)	63 (43.2)
Great	22 (16.7)	26 (21.8)	25 (17.1)
Really great	15 (11.4)	23 (19.3)	20 (13.7)
**Sexual desire**			
Never or almost never	112 (49.3)	123 (54.2)	93 (41)
Sometimes	64 (28.2)	61 (26.9)	69 (30.4)
Half of the time	31 (13.7)	29 (12.8)	40 (17.6)
Most of the time	16 (7.0)	13 (5.7)	17 (7.5)
Always or almost always	4 (1.8)	1 (0.4)	8 (3.5)

* At post-treatment a past week version of the SFQ was used.

### Effect of treatment on PTSD symptoms

The mean post-treatment CAPS-5 score of the sample was 16.46 (*SD* = 14.26). A significant decrease in CAPS-5 scores from pre- to post-treatment was found (*t* [227] = 27.92, *p* < 0.001) showing a large effect size (Cohen’s *d* = 2.30). The total PTSD remission rate after 8 days of treatment was 49.8%, whereas the proportion of patients with a loss of diagnosis was 75.3%.

### Sexual functioning following treatment

**Figure 2:**
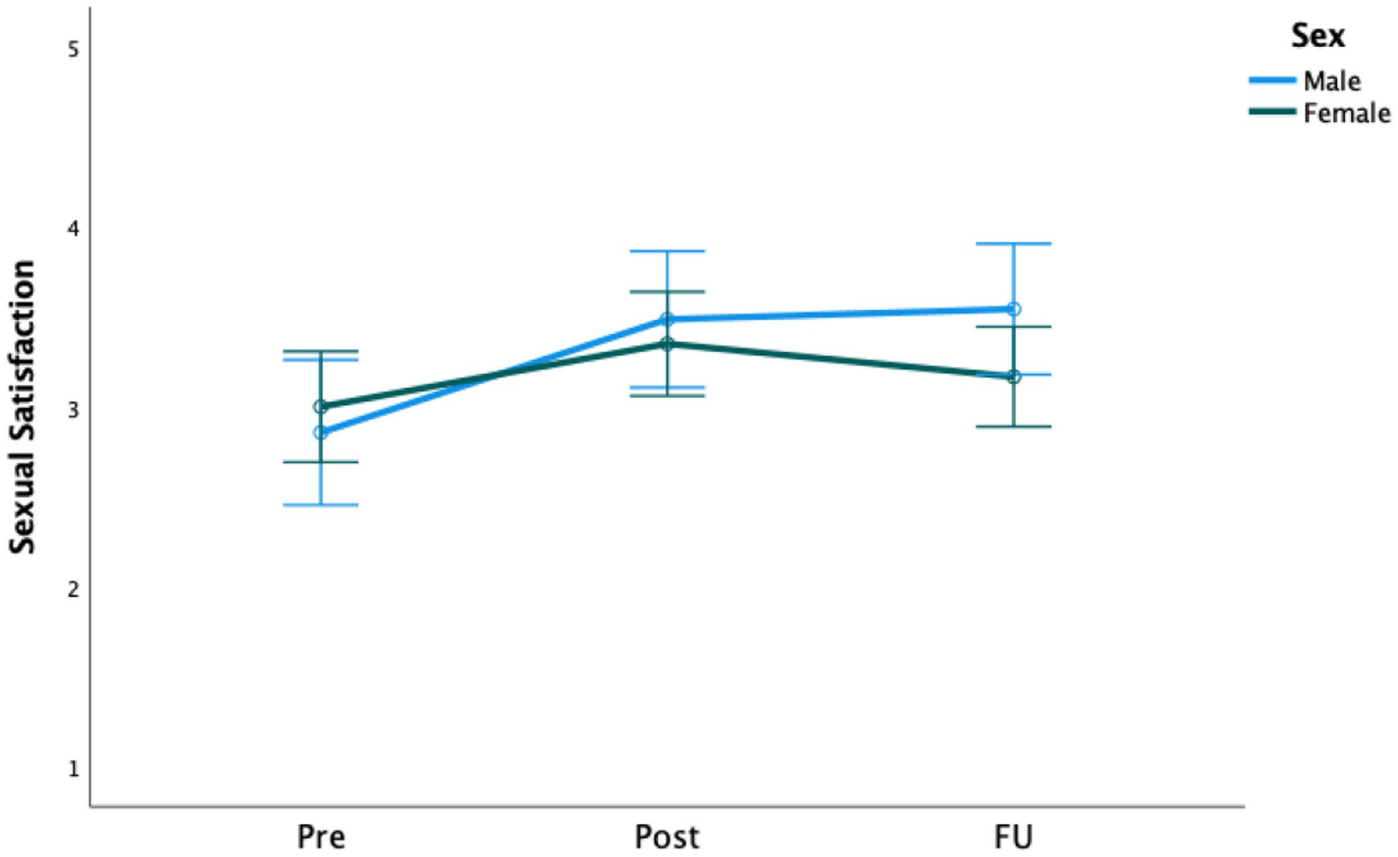
Treatment effect on sexual satisfaction for both men and women.

**Figure 3:**
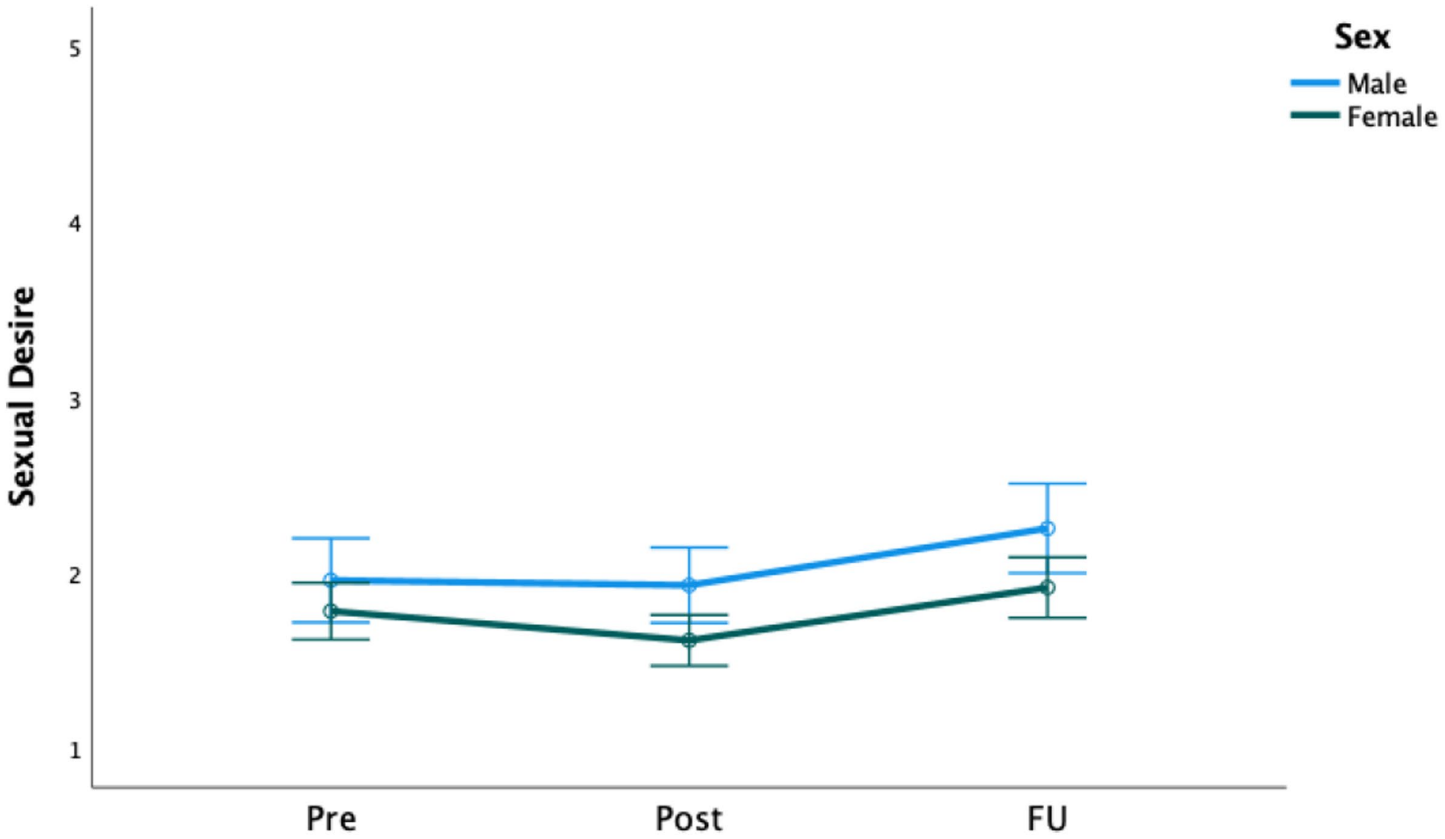
Treatment effect on sexual desire for both men and women.

Significant increases over time were found for both sexual satisfaction (*F* [2, 188] = 9.50, *p* < 0.001) and sexual desire scores (*F* [2, 452] = 11.39, *p* < 0.001; see Figures 2, 3). Regarding sexual desire, pairwise comparisons showed no changes from pre-treatment to 1 week following the termination of the treatment program (*p* = 0.142), but the scores changed significantly from both post-treatment to follow-up (*p* < 0.001) and from pre-treatment to follow-up (*p* = 0.015)*.* Descriptives and effect sizes (Cohen’s *d*) are presented in Tables 2, 3, respectively.

**Table 3:** Effect sizes of sexual functioning.

		Pre-treatment		Post-treatment		Follow-up		Effect size Pre-Post	Effect size Pre-FU
		Mean	*SD*	Mean	*SD*	Mean	*SD*	Cohen’s *d*	Cohen’s *d*
Sexual satisfaction	Total (*N* = 95)	2.95	1.20	3.40	1.12	3.31	1.09	0.32	0.39
	Men (*N* = 35)	2.86	1.35	3.49	1.22	3.54	1.07	0.49	0.56
	Women (*N* = 60)	3.00	1.11	3.35	1.07	3.17	1.09	0.32	0.15
Sexual desire	Total (*N =* 227)	1.84	1.02	1.71	0.93	2.02	1.10	*	0.17
	Men (*N* = 71)	1.96	1.10	1.93	0.95	2.25	1.13	*	0.26
	Women (*N = 156*)	1.78	0.99	1.63	0.91	1.92	1.07	*	0.14

* There was no significant effect.

### The relationship between gender and sexual functioning

There were no significant interaction effects from pre- to post-treatment and the 6-months follow-up of gender on sexual satisfaction (*F* [2, 186] = 2.66, *p* = 0.072) or desire (*F*[2, 450] = 0.77, *p* = 0.462), as shown in Figures 2, 3. However, tests of between-subjects effects showed a significant main effect of gender on sexual desire (*F* [1, 225] = 5.25, *p* = 0.023), indicating that, on average, men scored higher than women on sexual desire but not on sexual satisfaction. The effect sizes are presented in Table 3.

### The relationship between remission of PTSD and sexual functioning

No significant interaction effects of time (pre- to post-treatment and 6-month follow-up) and post-treatment remission on sexual satisfaction (*F* [2, 186] = 2.89, *p* = 0.058) or desire (*F* [2, 450] = 1.64, *p* = 0.195) were found. However, tests of between-subject effects showed significant main effects of remission for both sexual satisfaction (*F* [1, 93] = 4.74, *p* = 0.032) and sexual desire (*F* [1, 225] = 22.76, *p* < 0.001). This indicates that those with posttreatment PTSD remission generally reported higher levels of sexual functioning than those not in remission.

### The relationship between effect of the treatment on PTSD symptoms and sexual functioning at follow-up

Multiple linear regression analyzes were conducted to determine whether CAPS-5 score changes associated with treatment predicted follow-up sexual functioning scores, if controlled for pre-treatment sexual functioning scores. Significant regression equations were found for sexual satisfaction (*F* [2, 116)] = 22.96, *p* < 0.001), with an adjusted *R*^2^ of 0.27, and for sexual desire (*F* [2, 224] = 55.69, *p* < 0.001, with an adjusted *R*^2^ of 0.33. However, CAPS-5 change scores did not significantly predict sexual satisfaction (*β* = 0.006, 95% CI [−0.006, 0.017]; *t* [116] = 0.98, *p* = 0.328) or sexual desire (*β* = 0.005, 95% CI [−0.004, 0.013]; *t* [224] = 1.05, *p* = 0.293) at 6-months follow-up.

## Discussion

To the best of our knowledge, studies investigating the effects of trauma-focused treatment on self-reported sexual functioning have only been conducted among female patients with PTSD (Resick et al., 2003; O'Driscoll and Flanagan, 2016; Wells et al., 2019). The results of the present study partially support our hypothesis in that while men reported greater sexual desire overall, significant but small improvements in sexual functioning (i.e., both sexual satisfaction and sexual desire) for both men and women were found 6 months after treatment. Although after 8 days of treatment (i.e., 16 sessions of trauma-focused treatment) about half of the participants were in remission of PTSD, changes in PTSD symptoms were not found to be predictive of patients’ level of sexual satisfaction or sexual desire six months after treatment.

Our finding that both sexual satisfaction and sexual desire significantly improved with the application of trauma-focused treatment is in line with two previous studies (Resick et al., 2003; Wells et al., 2019), although an improvement in sexual desire could not be detected immediately following treatment but only several months later. One possible explanation is that patients were too exhausted immediately after the program to desire sexual contact, and therefore, more time may be required for this to improve. However, the finding that changes in PTSD symptoms were not predictive of the level of sexual satisfaction or sexual desire 6 months after treatment makes it unlikely that these changes can be explained by a reduction in the severity of PTSD symptoms *per se*. In this respect, our findings align with previous studies on sexual problems (O'Driscoll and Flanagan, 2016), but are at odds with those showing a significant relationship between the severity of PTSD and the presence of sexual dysfunction. It is important to note that not only did these latter studies focus on a specific subgroup of individuals suffering from PTSD (i.e., veterans; Schnurr et al., 2009; DiMauro et al., 2018; Letica-Crepulja et al., 2019), Letica-Crepulja et al. (2019) found in their study that not overall PTSD severity, but the severity of the D-symptom cluster (negative alterations in cognition and mood) determines the severity of sexual dysfunction. Therefore, we were interested in whether changes in the D-symptom cluster of PTSD from pre- to post-treatment were predictive of sexual functioning at the follow-up. However, this was not the case. Similarly, a recent network analysis of PTSD in patients with childhood sexual abuse (a patient group highly comparable to our patient sample) showed that sexual symptoms were relatively independent of PTSD symptoms, which could explain why a decrease in PTSD symptoms during trauma-focused treatment is not predictive of a decrease in sexual problems. Instead, other factors such as depression and hyperarousal symptoms (E-symptom cluster) were found to be more strongly related to sexual problems (Kratzer et al., 2022). In our own data, changes in the E-symptom cluster of PTSD did not prove to be a significant predictor of sexual functioning at the follow-up either. Additionally, since the use of psychoactive drugs is known to negatively influence sexual functioning, it is possible that in the 6 months following our treatment program, the prescription of patients’ psychoactive drugs changed and improved sexual functioning accordingly. Future research is needed to determine whether and to what extent other factors are responsible for improvements in sexual functioning and desire. A final alternative explanation for the improvements in sexual functioning and desire could be that other elements within the treatment program, such as participating in physical activities, had an enhancing effect on sexual satisfaction in the present study (Stanton et al., 2018). A randomized controlled trial showed that the contribution of physical activities to the other components of the intensive treatment program is negligible (Voorendonk et al., 2022), but the extent to which this contributed to patients’ sexual functioning had not been investigated.

The present study has several strengths. An important factor is the availability of longitudinal information from a large group of participants, both men and women, diagnosed with PTSD within a broad age range (18 to 72 years) and over a period of 6 months. Second, this study is unique in that every patient received exactly the same treatment consisting of two fully protocolled evidence-based trauma-focused therapies, with all patients receiving the same dose in terms of the number and length of therapy sessions. The fact that the therapy was carried out by a rotating team of therapists, with each session of the 16 sessions the patient received performed by a different therapist, may have had a potentially positive effect on potential bias and confounding factors. Third, the diversity of the sample regarding the nature of traumatic experiences enhances the generalizability of the findings. To our knowledge, this is the first study to investigate the effect of intensive trauma-focused treatment on sexual functioning, showing that treatment of PTSD could also have an impact when such treatment does not last longer than 2 weeks. As in most studies, several limitations of the present study should be acknowledged. First, our study lacked a control group, making it difficult to rule out the possibility that the observed improvements in sexual functioning could (in part) be attributed to the effect of time. Second, regarding the frequency with which prolonged exposure and EMDR therapy were applied, the generalizability of our findings to other longer-lasting (trauma-focused) treatment programs with lower frequencies of treatment sessions may be limited. Third, we were faced with missing data, as approximately one-third of the participants were not available or were unwilling to participate in the follow-up measurement due to various personal reasons. Missing data could lead to bias, meaning that the effect of the treatment could be underestimated or overestimated. However, the baseline comparisons did not show significant differences between the participants who were included and those who were excluded because of missing data. Finally, the SFQ was constructed for the purpose of this study and, therefore, is a limited measure of sexual functioning, as it does not encompass all aspects of sexual functioning, such as pain during sexual intercourse, orgasmic dysfunction, erectile dysfunction, and lubrication difficulties (American Psychiatric Association, 2013). Accordingly, it cannot be ascertained whether the total scores on this measurement represent the actual sexual functioning of the participants, which makes it difficult to compare the scores with those of other studies. Conversely, the items of the SFQ used in this study are similar to those of the Female Sexual Functioning Index (FSFI) and the International Index of Erectile Function (IIEF), which are both widely used and validated measures of sexual functioning (Rosen et al., 1997, 2000). Further research into the effects of trauma-focused treatments on sexual dysfunction in PTSD patients should be conducted to determine which aspects of sexual functioning are most severely impaired in PTSD patients and, thereby, also consider sexual dysfunctions other than impaired sexual satisfaction and desire.

In conclusion, the results of the present study suggest that sexual satisfaction and desire are likely to improve following intensive trauma-focused treatment for both men and women, without specifically addressing sexual functioning. However, these improvements were small, and for sexual desire, this effect was only found at the 6-month follow-up. Importantly, these effects did not seem to depend on the remission of PTSD or on the relative changes in PTSD symptoms associated with treatment. Clinical implications indicate that, although small improvements in sexual functioning can be found after intensive trauma-focused treatment, it may be worthwhile to add interventions specifically aimed at the improvement of sexual functioning to further enhance positive results. Nevertheless, replication and further research using a randomized controlled design, including several other relevant factors for sexual functioning, such as comorbid mood disorders, use of psychoactive drugs, and self-esteem, are needed before firm conclusions can be drawn.

## Data availability statement

The raw data supporting the conclusions of this article will be made available by the authors after request to the first author, CW, c.vanwoudenberg@psytrec.nl.

## Ethics statement

This study was performed in accordance with the principles of the Declaration of Helsinki. According to the Medical Ethics Review Committee of VU University Medical Centre (registered with the US Office for Human Research Protections (OHRP) as IRB00002991, FWA number FWA00017598), the Medical Research Involving Human Subjects Act (WMO) does not apply to this study. For this reason, official approval of this study by the committee was not required. The patients/participants provided their written informed consent to participate in this study.

## Author contributions

CW, EV, BT, VB, LR, AM, and AJ contributed to conception and design of the study. LR organized the database. CW performed the statistical analysis. VB, BT, and CW wrote the first draft of the manuscript. AJ, EV, and AM wrote sections of the manuscript. All authors contributed to the article and approved the submitted version.

## Conflict of interest

AM receives income from published book chapters on PTSD and from the training of postdoctoral professionals with prolonged exposure. AJ received income from published books on EMDR therapy and from the training of postdoctoral professionals in this method.

The remaining authors declare that the research was conducted in the absence of any commercial or financial relationships that could be construed as a potential conflict of interest.

The reviewer DF declared a shared parent affiliation with the author AJ to the handling editor at the time of review.

## Publisher’s note

All claims expressed in this article are solely those of the authors and do not necessarily represent those of their affiliated organizations, or those of the publisher, the editors and the reviewers. Any product that may be evaluated in this article, or claim that may be made by its manufacturer, is not guaranteed or endorsed by the publisher.
