# Supplementary material for: The impact of intensive trauma-focused treatment on sexual functioning in individuals with PTSD
Source: Front Psychol. 2023 Aug 8;14:1191916. doi: 10.3389/fpsyg.2023.1191916 (PMC10442952; doi:10.3389/fpsyg.2023.1191916)
Supplement: Supplementary file 1 [file Data_Sheet_1.doc]

# Appendix I

Seksueel functioneren vragenlijst (SFV), Nederlandse versie (pre-treatment versie)

Welkom bij deze vragenlijst.

Uit eerder onderzoek blijkt dat sommige mensen met PTSS problemen hebben met hun seksueel functioneren, en daar veel last van hebben. Om die reden leggen wij u een viertal vragen voor over de samenhang tussen PTSS-klachten en uw functioneren op seksueel gebied. Ook de antwoorden op deze vragen worden geanonimiseerd en vertrouwelijk verwerkt.

1. Heeft u een vaste partner?


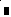
 Ja


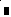
 Nee

2. Hoe vaak had u in de afgelopen vier weken geslachtsgemeenschap of heeft u andere seksuele handelingen verricht (waaronder zelfbevrediging)?


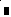
 Geen enkele keer (*ga verder met vraag 4*).


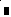
 1-2 keer (*ga verder met vraag 3*).


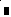
 3-4 keer (*ga verder met vraag 3*).


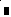
 5-6 keer (*ga verder met vraag 3*).


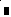
 7-10 keer (*ga verder met vraag 3*).


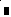
 11 keer of meer (*ga verder met vraag 3*).

3. In welke mate heeft u de afgelopen vier weken genoten van het hebben van geslachtsgemeenschap of andere seksuele handelingen (waaronder zelfbevrediging)?


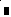
 Niet genoten


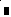
 Niet erg genoten


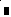
 Redelijk genoten


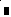
 Erg genoten


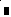
 Heel erg genoten

4. Hoe vaak had u de afgelopen vier weken seksuele verlangens?


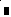
 Bijna nooit of nooit


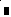
 Een paar keer (veel minder dan de helft van de tijd)


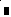
 Soms (ongeveer de helft van de tijd)


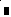
 Meestal (veel meer dan de helft van de tijd)


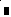
 Bijna altijd of altijd

Einde van de vragenlijst.

# Appendix II

Sexual Functioning Questionnaire, English version (pre-treatment version)

Welcome to this questionnaire.

Previous research has shown that patients with posttraumatic stress disorder (PTSD) may suffer from problems in sexual functioning, and that these problems may cause distress. The following four questions are aimed at determining the correlation between your PTSD-symptoms and sexual functioning. Your answers will be anonymized and processed confidentially.

1. Do you have a partner?


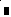
 Yes


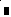
 No

2. How often did you have sexual intercourse or other sexual activities (including masturbation) in the past four weeks?


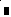
 None (*continue with question 4*).


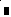
 1-2 times (*continue with question 3*).


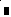
 3-4 times (*continue with question 3*).


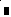
 5-6 times (*continue with question 3*).


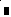
 7-10 times (*continue with question 3*).


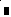
 11 times or more (*continue with question 3*).

3. To what degree did you enjoy having sexual intercourse or other sexual activities (including masturbation) in the past four weeks?


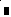
 No satisfaction


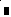
 Little satisfaction


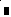
 Moderate satisfaction


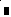
 Great satisfaction


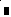
 Really great satisfaction

4. How often did you have sexual desire in the past four weeks?


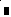
 Never or almost never


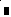
 Sometimes (less than half of the time)


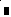
 Half of the time


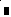
 Often (more than half of the time)


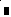
 Always or most of the time

End of the questionnaire.
